# TB research requires strong protections, innovation, and increased funding in response to COVID-19

**DOI:** 10.1186/s13063-021-05331-4

**Published:** 2021-05-29

**Authors:** B. T. Nyang’wa, A. N. LaHood, C. D. Mitnick, L. Guglielmetti

**Affiliations:** 1 Manson Unit, Médecins Sans Frontières, London, UK; 2 Clinical Research Department, London School of Hygiene & Tropical Medicine, London, UK; 3 Department of Global Health and Social Medicine, Harvard Medical School, Boston, MA 02115 USA; 4 Partners In Health, Boston, MA USA; 5 Médecins Sans Frontières, Paris, France; 6 Sorbonne Université, INSERM, U1135, Centre d’Immunologie Et Des Maladies Infectieuses, Paris, France; 7 APHP, Groupe Hospitalier Universitaire Sorbonne Université, Hôpital Pitié-Salpêtrière, Centre National De Référence Des Mycobactéries Et De La Résistance Des Mycobactéries Aux Antituberculeux, Paris, France

To the editor:

When 2020 opened, approximately 11 million new tuberculosis (TB) cases and nearly 1.5 million TB-related deaths were predicted during the year. And, the gap between required and available research and development resources for TB was estimated at more than 1 billion USD [1]. COVID-19, the global pandemic of disease caused by the novel severe acute respiratory syndrome coronavirus 2 (SARS-CoV-2), has surely widened that gap by worsening the TB pandemic. Worryingly, the economic devastation wrought by COVID-19 portends further dramatic reductions in funds for other health research [2], including for multidrug-resistant tuberculosis (MDR-TB). Such constraints will prevent the use of extant TB research infrastructure to support cross-disease benefits in the struggle against COVID-19 and future pandemics [3].

Several reports have estimated the impact of the COVID-19 pandemic on TB incidence and mortality. In one (conservative) scenario, a 3-month lockdown and 10-month period to restore TB services are estimated to cause 6.3 million excess TB cases and nearly 1.4 million excess TB deaths over 5 years [4]. Another recent model illustrated that even with presumed reduced social contact, the effects of service interruptions would still be profound, especially in settings, like China, in which reactivation is a major driver of disease burden [5]. Pandemic and containment effects conspire to worsen the TB pandemic in many ways. Health facilities have been shuttered or repurposed for COVID-19 care, leading to widespread reports of TB facility closures [6]. Drug shortages are expected as a result of supply-chain disruptions and reduced exportation [7]. TB laboratories activities are often impacted due to resource re-allocation and lack of trained staff [8]. Opportunities for TB transmission increase during shelter-in-place orders, curfews, and with reassignment of health staff to COVID-related activities. Meanwhile, protections against TB and poor outcomes are reduced with loss of wages and compromised nutrition [9]. The overall direct and indirect impact of COVID-19 on health, with important implications for TB, has been recently described [10].

This unprecedented constellation of factors heightens the importance of innovation in TB treatment. Even before COVID-19, a robust modelling exercise concluded that new interventions would be required to achieve the sustainable development goal target for TB in certain high-burden TB countries, such as India and China [11]. Such innovation emerges through the conduct of rigorous, carefully monitored, albeit lengthy, clinical research. An urgent research objective is the development of, effective, safe, well-tolerated, injectable-sparing, and shortened treatment for all forms of MDR-TB. Achievement of this goal is even more important in the era of COVID-19: regimens that demand less of patients and health systems, and that can assure better outcomes, are critical in times of disruption [12]. Yet COVID-19 pandemic conditions threaten, rather than accelerate, currently enrolling (and planned) TB trials. Some regulators and trial experts have recommended delaying or suspending clinical research until, for example, “the SARS-CoV-2 viral burden… is low” to avert SARS-CoV-2 infections and avoid compromising scientific integrity of studies [13]. It is critical to balance the human and scientific risks of maintaining health research in this environment with the health consequences of delays and disruptions.

Three trials affected by the COVID-19 pandemic, endTB, endTB-Q, and TB-PRACTECAL, all sponsored by Médecins Sans Frontières, illustrate how COVID-19 aggravates the well-documented inherent challenge of conducting MDR-TB treatment trials [14]. The disease is most prevalent in populations and health systems suffering from grave inequalities that amplify pandemic effects [14]. endTB (NCT02754765), endTB-Q (NCT03896685), and TB-PRACTECAL (NCT02589782) are late-stage multi-country, randomized, pragmatic, open-label clinical trials with internal, dynamic control arms. All feature innovative study designs: endTB includes Bayesian response-adaptive randomization; endTB-Q adapts treatment duration according to characteristics and treatment response of participants; TB-PRACTECAL is an adaptive multi-arm multi-stage trial. Each trial includes rifampin-resistant (RR) TB patients: endTB includes patients with fluoroquinolone (FQ)-susceptible RR-TB while endTB-Q includes FQ-resistant RR-TB patients; TB-PRACTECAL includes patients regardless of FQ resistance status. Two (endTB and TB-PRACTECAL) had been enrolling for an extended period prior to the outbreak of COVID-19. The third, endTB-Q, was activated early in the pandemic. endTB, endTB-Q, and TB-PRACTECAL planned to enrol 750, 324, and 630 participants from 7, 6, and 3 countries, respectively.

All three trials have had to adopt strategies to ensure patient and staff safety while maintaining study activities—including treatment—and integrity. Strategies include establishing alternate locations for in-person visits; increased use of remote, virtual visits including video observation for treatment monitoring; extending the intervals between visits and increasing the quantity of medications dispensed; and rethinking trial monitoring to conform to regulatory guidance [15]. Moreover, substantial investments are required to protect study participants and staff from risks of transmission of SARS-CoV-2. This includes testing for SARS-CoV-2 infection, provision of personal protective equipment, means (nutritional and social support, additional housing) to facilitate quarantine and/or isolation, and increased use of private transport to avert COVID-19 transmission risk conferred by public transport. Human resource adjustments have included staggered work schedules, (improved) paid medical leave for sick staff, and enhanced salaries and benefits to retain study staff in the face of increased risk and emerging, better-remunerated COVID-19-response job opportunities. Lastly, with fewer patients receiving TB services, trial enrollments and timelines have been impacted (see Table 1). endTB and endTB-Q results are expected to be delayed by 6–8 months in part due to important decreases in TB case notifications in Lesotho and Kazakhstan. TB-PRACTECAL’s expected completion was delayed by 12 months; the stage 1 to stage 2 adaptation that had been planned to start in March 2020 was deprioritized to focus on COVID-19 adaptations and was not started until October 2020. The trial has, however, terminated recruitment early following an Independent Data and Safety Monitoring Board’s recommendation unrelated to the delays [16].

**Table 1:** DR-TB background notifications and trial enrollments in select endTB, endTB-Q and TB-PRACTECAL study areas in 2019 and 2020, and percent change

		DR-TB notifications or trial enrollments			DR-TB notifications or trial enrollments		
		Quarter 1 (Jan–Mar)			Quarter 2 (Apr–Jun)		
Country	Setting	2019	2020	% change 2019–2020	2019	2020	% change 2019–2020
Belarus	Background notification^*^	253	232	− 8.3%	212	163	− 23.1%
	TB-PRACTECAL^†^	6	12	+ 100%	5	8	+ 60%
Kazakhstan	Background notification^‡^	95	62	− 34.7%	93	45	− 51.6%
	endTB AND endTB-Q	18	6	− 66.6%	19	6	− 68.4%
India	Background notification^§^	230	252	+ 9.57%	217	142	− 34.6%
	endTB AND endTB-Q^¶^	NA	NA	NA	NA	NA	NA
Lesotho	Background notification^**^	49	38	− 22.4%	38	21	− 44.7%
	endTB AND endTB-Q	6	1	− 83.3%	5	2	− 60%

^*^National data

^†^Trial catchment area was expanded in Q4 of 2019

^‡^Almaty and Nur-Sultan cities

^§^Centenary, Govandi, and Chembur districts in Mumbai

^¶^endTB and endTB-Q trials began enrolling in Q4 2020 in India

^**^National data

Budget projections for research studies underway could not have anticipated either the delays or the necessary protective measures [7]. Without increased commitments to TB research funding, critical innovations in TB treatment will be compromised; opportunities are lost to leverage learning from application of these mitigation measures to routine TB care, and about joint COVID and TB services [3].

In summary, the COVID-19 pandemic threatens populations through myriad direct and indirect effects. Disrupted TB services and research figure prominently among the ways in which COVID-19 could leave a lasting legacy. It is more critical than ever to increase funding for TB research to enhance the ability to prevent, diagnose, and treat TB in good times and in bad.

## Data Availability

Not applicable.
